# Construction of viral protein-based hybrid nanomaterials mediated by a macromolecular glue†

**DOI:** 10.1039/d2tb02688k

**Published:** 2023-06-06

**Authors:** Shuqin Cao, Sandro Peeters, Sandra Michel-Souzy, Naomi Hamelmann, Jos M. J. Paulusse, Liu-lin Yang, Jeroen J. L. M. Cornelissen

**Affiliations:** a Laboratory for Biomolecular Nanotechnology, MESA+ Institute for Nanotechnology, University of Twente P.O. Box 217 7500 AE Enschede The Netherlands J.J.L.M.Cornelissen@utwente.nl; b State Key Laboratory of Oral Diseases, West China Hospital of Stomatology, Sichuan University Chengdu 610041 China; c College of Chemistry and Chemical Engineering, Xiamen University 361005 Xiamen China llyang@xmu.edu.cn

## Abstract

A generic strategy to construct virus protein-based hybrid nanomaterials is reported by using a macromolecular glue inspired by mussel adhesion. Commercially available poly(isobutylene-*alt*-maleic anhydride) (PiBMA) modified with dopamine (PiBMAD) is designed as this macromolecular glue, which serves as a universal adhesive material for the construction of multicomponent hybrid nanomaterials. As a proof of concept, gold nanorods (AuNRs) and single-walled carbon nanotubes (SWCNTs) are initially coated with PiBMAD. Subsequently, viral capsid proteins from the Cowpea Chlorotic Mottle Virus (CCMV) assemble around the nano-objects templated by the negative charges of the glue. With virtually unchanged properties of the rods and tubes, the hybrid materials might show improved biocompatibility and can be used in future studies toward cell uptake and delivery.

## Abstract 2

A dopamine based macromolecular glue is used to facilitate the construction of hybrid nanomaterials that are coated with virus capsid proteins, with the aim to improve stability, biocompatibility, and function.

## Graphical abstract

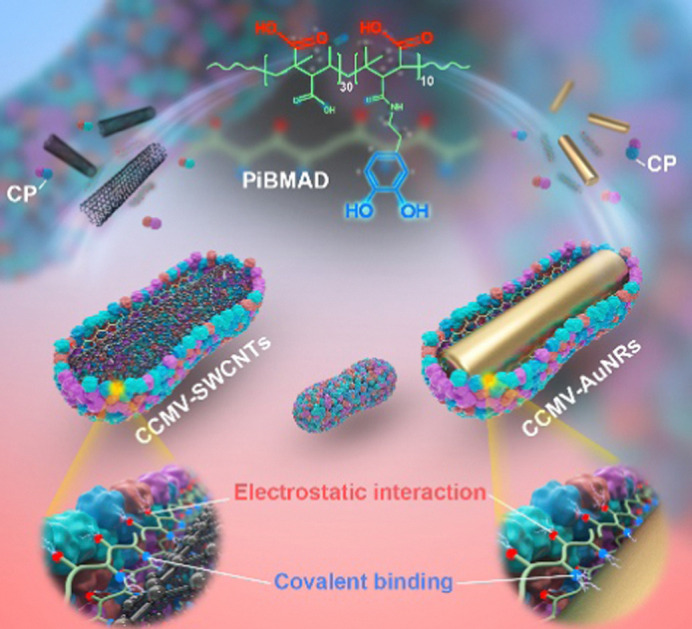

## Introduction

Nanomaterials have found numerous applications in catalysis,^1,2^ biosensors,^3^ drug delivery^4^ and bioimaging^5,6^ due to their controlled chemical compositions and structures, large surface-to-volume ratios, various functional groups, and surfaces. Among them, protein cages, such as virus-based hybrid nanomaterials, show interesting properties in electronics,^7^ catalysis,^8^ drug/gene delivery,^9,10^ imaging and immune-therapy,^11^ that are largely based on their reversible assembly, uniform size, facile modification, and capability of functional cargo encapsulation.^12,13^

These functional protein-organic/inorganic hybrid nanomaterials are prepared *via* self-assembly of proteins,^12,14–17^ chemical conjugation,^5,18^ and protein immobilization.^19^ Protein cages derived from the Cowpea Chlorotic Mottle Virus (CCMV) have been widely used as nanocarriers with various cargos such as polystyrene sulfonate (PSS),^20^ oligonucleotides,^21^ functionalized enzymes,^22^ gold nanoparticles,^23^ and luminescent materials.^14^ These hybrid materials combine efficiently the biocompatibility of the protein coating with the functionality of the cargo, as exemplified by our recent study on nanodiamonds for intracellular trajectory analysis.^24^ It is, however, crucial in the formation of these hybrid materials to maintain the functional properties of the nanomaterials. The capsid protein of CCMV is capable of self-assembly into empty capsids at pH 5 with high salt concentration, when the main driving force is the interaction between capsid proteins. While for efficient loading, negatively charged cargo surfaces are generally required to guarantee strong electrostatic interactions between the capsid proteins and the desired cargos at neutral pH. Therefore, for diverse nanometer-sized cargo candidates, such as silicon quantum dots, gold nano-objects and carbon nanotubes with neutral surfaces, a surface modification either by coupling oligonucleotides,^6^ PSS or thiol-based ligands is a prerequisite.^7^ However, this surface modification usually involves tedious organic synthesis, meanwhile, it suffers from the aggregation of these nano-objects that lead to instability and the loss of intrinsic properties. To tackle this problem, we propose a generic adhesive material that can serve as an interfacial molecular glue to facilitate the coating of nanometer-sized cargo with CCMV capsid proteins, to further expand the toolbox for the colloidal stabilization, biocompatibility and eventual cellular uptake of functional nanomaterials.^24^

The structural design and core capabilities of this molecular glue should meet two requirements: (i) as an adhesive material, it can intimately interact with a wide range of nanomaterial surfaces; and (ii) as a template to induce the coating of capsid proteins on nanomaterials, it should incorporate negatively charged functional groups (i.e, carboxylic acids, sulfates, phosphates). Catechol functionalized polymers inspired by natural mussels have been developed as tissue adhesives for bio-applications^25–27^ Since the catechol group is capable of having strong non-covalent interactions with metal ions, polarized organic groups, or covalent crosslinking *via* oxidation.^26,28–30^ Hence, the catechol group is an ideal building block to meet the first requirement for constructing the molecular glue. Meanwhile, commercially available poly(isobutylene-*alt*-maleic anhydride) that inherently contains multiple carboxyl groups can serve as the template building block to meet the second requirement. Therefore, we prepared catechol modified poly(isobutylene-*alt*-maleic anhydride) as the molecular glue for constructing the desired nanomaterials.

## Results and discussion

**Fig. 1:**
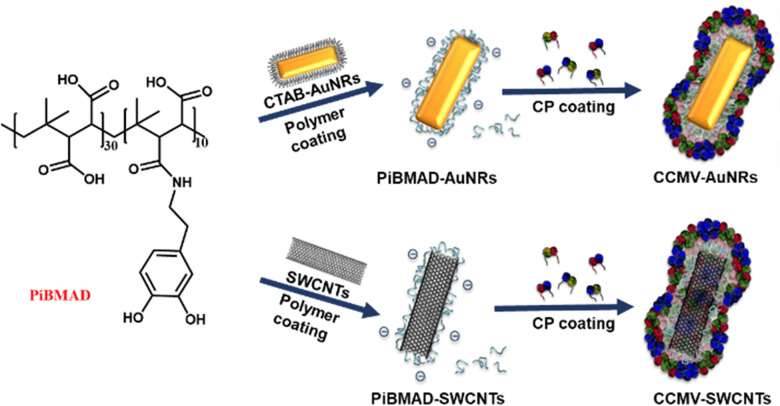
Schematic representation of mussel-inspired molecular glue to induce the coating of two nanoobjects, AuNRs and SWCNTs by CCMV capsid proteins. Both the AuNRs and SWCNTs were first coated with random functionalized PiBMAD, then CCMV capsid proteins were coated on their surface through electrostatic interactions.

The molecular glue was synthesized by amination of poly(isobutylene-*alt*-maleic anhydride) with dopamine (PiBMAD).^31^ Dopamine was used to introduce the catechol group for the adhesiveness of this generic molecular glue, as it is a well-known molecular mimic of mussel foot protein.^32,33^ As a proof of concept, two representative nanomaterials, gold nanorods (AuNRs) and single-walled carbon nanotubes (SWCNTs), have been selected as cargo candidates to verify the applicability of this molecular glue (Fig. 1).

**Fig. 2:**
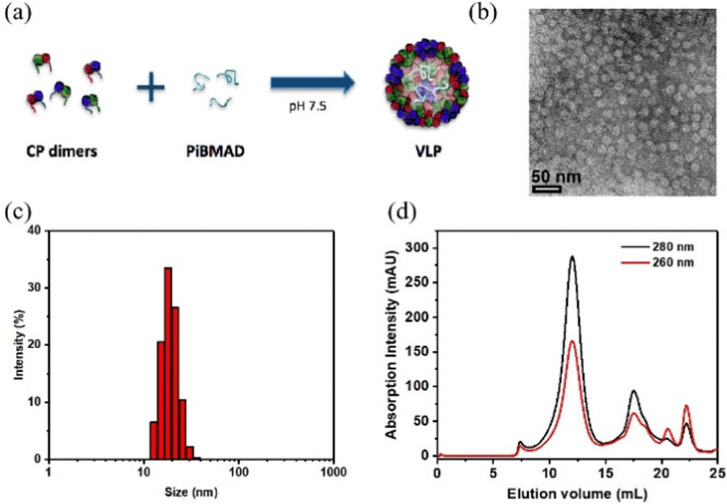
Preparation of PiBMAD scaffolded virus-like particles. (a). Schematic representation of PiBMAD induced formation of virus-like particles; (b). TEM mages of formed virus-like particles; (c). DLS data shows well distributed virus-like particles sized around 18 nm; (d). FPLC analysis of virus-like particles templated by PiBMAD.

PiBMAD was synthesized as described in the ESI† and Fig. S1a (ESI†). Commercially available PiBMA with an average molechular weight (*M*_w_) of 6 kDa was used (Fig. S1b, ESI†). To maintain water solubility of the polymer and keep sufficient negative charges, around 12.5% of the available carboxylate groups were equipped with dopamine according to ^1^H-NMR analysis (Fig. S1c, ESI†). The capability of PiBMAD to induce the assembly of capsid proteins was first checked by incubating the proteins in a buffer with the polymer at pH 7.2 (see ESI† for details). The formation of virus-like particles was evident and purified from fast protein liquid chromatography (FPLC) equipped with a UV-vis detector (Fig. 2d). The absorption data showed an overlapping signal at two detection wavelengths (*λ* = 260 nm and 280 nm) at an elution volume of *V* ≈ 11 mL, whereas excess capsid protein eluted out at a larger elution volume (Fig. S8, ESI†) indicating the formation of virus-like particles. The fraction was isolated and analyzed by transmission electron microscopy (TEM) and dynamic light scattering (DLS) (Fig. 2b and c). TEM analysis revealed the formation of spherical nanoparticles with an average diameter of 19 nm. DLS showed that the isolated fraction contained nanoparticles with a diameter of 18.0 ± 3.6 nm, which is consistent with the TEM results. These data are in line with the previously confirmed *T =* 1 icosahedral symmetry for CCMV based virus-like particles formed at neutral pH on a polyanionic template.^34^

**Fig. 3:**
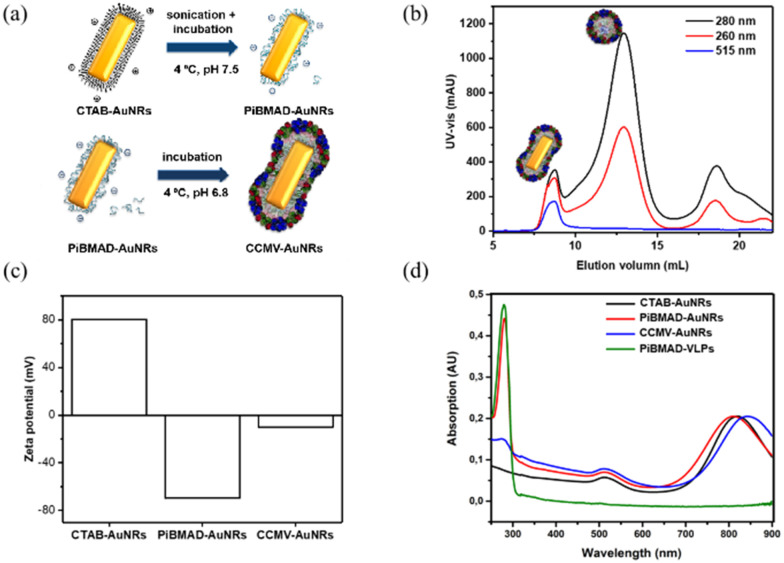
(a). Schematic presentation the formation of CCMV-AuNRs mediated by PiBMAD; (b). FPLC analysis of CCMV-AuNRs; (c). Changes of zeta potential of AuNRs during the different coating steps; (d). UV-vis spectra of original CTAB-AuNRs, PiBMAD-AuNRs, CCMV-AuNRs, and PiBMAD-virus-like particles.

Gold nanorods (AuNRs) belong to an interesting class of nanosized objects for a plethora of biomedical and biotechnological applications such as sensing, imaging and others. However, these applications rely on their stability in biological fluids, avoiding aggregation and precipitation. Surface coating of gold nanorods with biocompatible proteins may facilitate the bioapplications of these nanomaterials. To this end, surface coating of AuNRs by CCMV proteins was successfully realized by PiBMAD scaffolding (Fig. 3a), detailed experimental methods are described in the ESI.† The coated AuNRs were analyzed by FPLC (Fig. 3b) monitoring the elution at *λ* = 260, 280 and 515 nm. The elution volume of the first fraction around 7.5 mL, of all three coinciding wavelengths, is smaller than the typical elution volume of virus-like particles (11 mL), suggesting the formation of larger particles. Other than that, the transverse plasmon resonance peak of AuNRs at *λ* = 515 nm was also and only detected in the first fraction, confirming that this fraction contains coated gold nanorods (CCMV-AuNRs).^35^

Zeta potential measurements were carried out to check the change of surface charge of AuNRs during coating (Fig. 3c). When the AuNRs were first coated by PiBMAD, the zeta potential of AuNRs reversed from positive to negative, due to the abundant carboxyl groups of polymer chains. When finally coated by capsid proteins, as expected, the zeta potential of CCMV-AuNRs increased from −75 mV to −10 mV. The longitudinal plasmon resonance peak (LPRW) of the original AuNRs was located at *λ* = 808 nm, after coating by PiBMAD, the LPRW of PiBMAD-AuNRs was slightly shifted to *λ* = 805 nm, while after coating by capsid proteins, a 50 nm red shift of the longitudinal plasmon resonance (Fig. 3d) was observed.

**Fig. 4:**
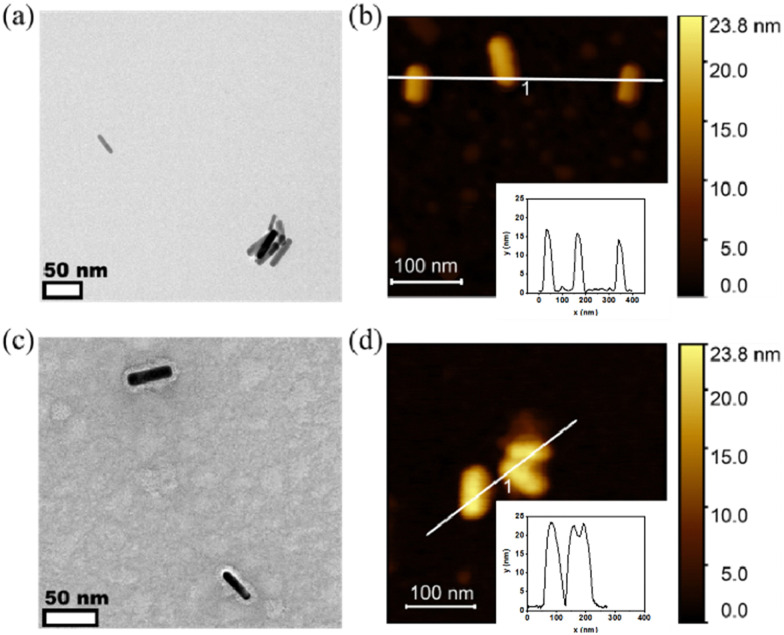
TEM and AFM analysis of PiBMAD-AuNRs and CCMV-AuNRs. (a) Negative stained TEM image of PiBMAD-AuNRs indicates a size around 10 × 40 nm; (b) AFM image of PiBMAD-AuNRs, insert height analysis of PiBMAD-AuNRs; (c) negative stained TEM image of CCMV-AuNRs showing the protein layer (thickness around 5 nm); (d). AFM image of CCMV-AuNRs, insert height analysis of CCMV-AuNRs; for the PiBMAD-AuNRs, the height is around 15 ± 2.3 nm which demonstrates around 2.5 nm coating of PiBMAD; while the height of CCMV-AuNRs is around 25 nm, which indicates around 5 nm coating of capsid proteins, this is in line with the expected capsid thickness.

The morphology of PiBMAD-AuNRs and CCMV-AuNRs were studied by transmission electronic microscopy (TEM) and atomic force microscopy (AFM). Both TEM and AFM analysis showed that the morphology of AuNRs was unchanged after coating with PiBMAD (Fig. 4a and b). The original height of AuNRs was 10 nm, while the overall height of PiBMAD-AuNRs was around 15 nm (Fig. 4b insert), suggesting a ∼2.5 nm thickness of polymer layer. The thickness of capsid proteins layer on CCMV-AuNRs is estimated to be ∼5 nm (Fig. 4c and d), which is consistent with the thickness of the native CCMV capsid shell, suggesting a monolayer coating of capsid proteins on the AuNRs surface. AFM micrograph and height analysis on single PiBMAD-AuNRs and CCMV-AuNRs further supports this conclusion (Fig. S5, ESI†).^36,37^ According to the literature^37^ and our investigation, we conclude that capsid proteins formed subunits such as heximers and pentamers while coating on the surface of AuNRs, and defects can be seen from Fig. 4c.

To avoid the aggregation of AuNRs during the coating process, an optimized concentration of PiBMAD is required (Fig. S2, ESI†). The strongest LPRW absorption peak of PiBAMD-AuNRs suggests minimal aggregation when the PiBMAD concentration is 3 mg mL^−1^. This is explained by less catechol being attached to the surface of AuNRs at lower concentrations of PiBMAD and when the concentration of PiBMAD is too high, catechol groups of PiBMAD might cause inter-nanoparticle crosslinking.^38,39^ It should be further noted that the buffer solution (*i.e*, salt concentrations, pH) also plays an important role in coating capsid proteins onto the AuNRs (Fig. S3, ESI†). FPLC data (Fig. S3a, ESI†) indicates a thicker layer of capsid proteins is coated on AuNRs at pH 6.8 compared to pH 7.2, which is in line with a higher UV absorption at *λ* = 280 nm by capsid proteins (Fig. S3b, ESI†).

Because of the large surface area, excellent chemical stability and rich polyaromatic structure, carbon nanotubes (CNTs) are an emerging nanomaterial as scaffolds for biosensors^40–42^ and anticancer drug delivery cargos.^43^ The main challenge of CNTs as biomaterials, is their poor aqueous solubility due to their high hydrophobicity. To improve the aqueous solubility, the functionalization of CNTs with biocompatible and hydrophilic functional organic species are the general approach.^44–46^ Herein, we demonstrate a facile protein coating strategy on single-walled carbon nanotubes (SWCNTs) by employing the molecular glue PiBMAD without modification of SWCNTs. The detailed coating process is described in the ESI.†

**Fig. 5:**
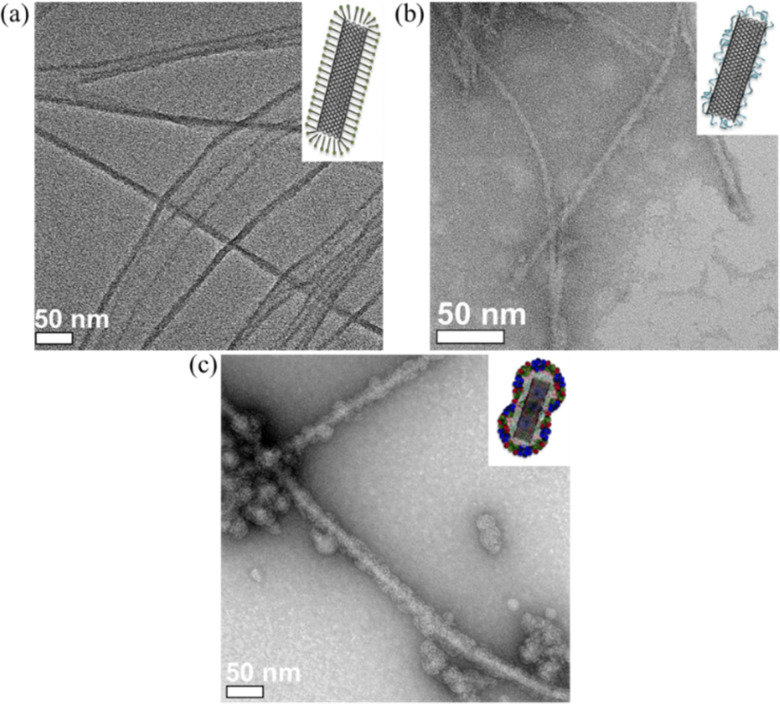
TEM images of (a) SDS-SWCNTs, (b) PiBMAD-SWCNTs, and (c) CCMV-SWCNTs.

SWCNTs dispersed with sodium dodecyl sulfate (SDS) showed few bundles (Fig. 5a). After ligand exchange with PiBMAD, the size and distribution of SWCNTs were almost unchanged (Fig. 5b). While after coating with capsid proteins, the average diameter of CCMV-SWCNTs was around 20 nm (Fig. 5c, Fig. S7, ESI†), suggesting a protein monolayer with ∼5 nm thickness on the surface of SWCNTs. Raman spectroscopy (Fig. S4, ESI†) also confirmed the presence of SWCNT before and after coating with PiBMAD, based on the unchanged characteristic peaks associated with CNT.

In control experiments, other ligands such as bis(*p*-sulfonatophenyl)phenylphosphine dihydrate dipotassium salt (BSPP) and poly-dopa were used as templates for CCMV capsid proteins coating (Fig. S6, ESI†). For the AuNRs, both BSPP and PiBMAD templated the formation of CCMV-AuNRs and an apparent single layer of capsid proteins coating was observed, while poly-dopa templated coating induced aggregation of AuNRs which might be caused by crosslinking of AuNRs by the poly-dopa (Fig. S6a–c, ESI†). With respect to SWCNTs, only PiBMAD induced the formation of well-dispersed CCMV-SWCNTs (Fig. S6d–f, ESI†). These results are in line with the concept of using PiBMAD as a generic molecular glue for the construction of viral protein coated hybrid nanomaterials.

**Fig. 6:**
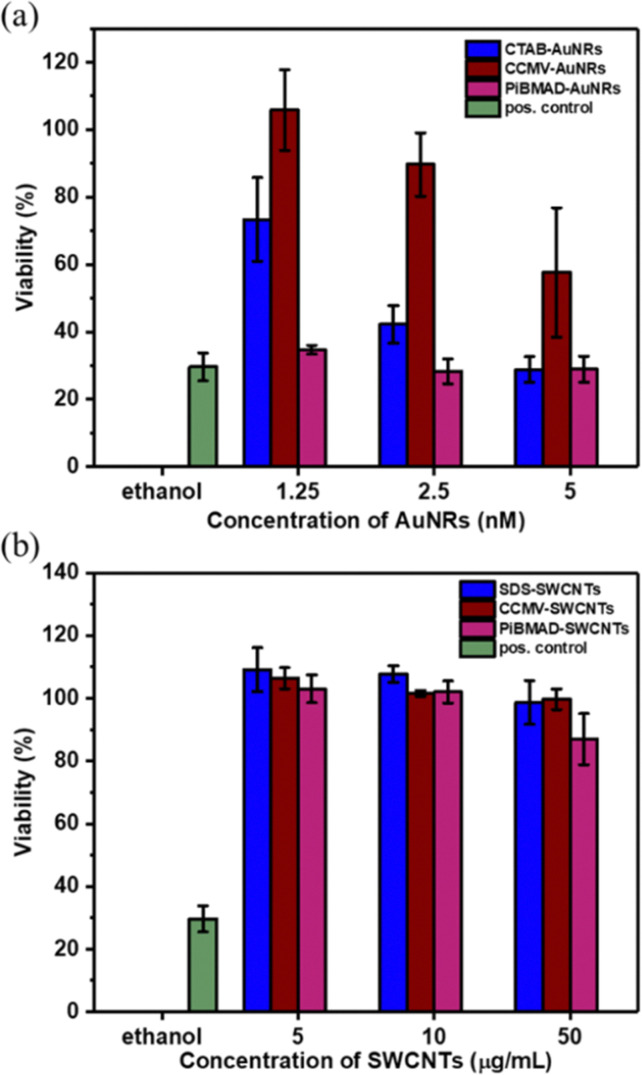
Cell cytoxicity of AuNRs and SWCNTs at various concentration. (a). Cell viability of AuNRs at various concentration (1.25–5 nmol L^−1^); (b). cell viability of SWCNTs at various concentration (5–50 μg mL^−1^).

Increasing the colloidal stability of functional nanomaterials can be achieved by a variety of chemical means. We chose, however, the biorelevant coating of virus capsid proteins to potentially improve biocompatibility and eventually direct cell uptake.^10,24^ To demonstrate the compatibility of CCMV-AuNRs and CCMV-SWCNTs for potential bio-applications, cytotoxicity studies were carried out at various concentrations of the biohybrid materials in HeLa cells. As shown in Fig. 6a, the cells incubated with CCMV-AuNRs revealed significantly higher viability compared to CTAB-AuNRs and PiBMAD-AuNRs over the tested concentration range (from 1.25 to 5 nmol L^−1^), although a concentration dependent cytotoxicity was shown for all three samples. The SWCNTs with different surface coatings demonstrate similar cell viability at the various concentrations studied (range from 5–50 μg mL^−1^) (Fig. 6b). Both AuNRs^47^ and CCMV^48^ based nanostructures are presumed to be taken up in HeLa cells by clathrin mediated endocytose. The different surface chemistry of the three samples in Fig. 6a, with CTAB-AuNRs being positive and CCMV-AuNRs only slightly negatively charged, likely accounts for the differences in cytotoxicity. In the case of the SWCNT based samples the marginal difference in cell viability might be attributed to limited uptake of these samples by the cells. For both classes of nanomaterials, further and more detailed studies are needed to understand the interactions with cells. It is clear, however, that the successful coating with capsids proteins allows for targeted modification of the surface, for example, with proteins equipped with cell penetrating peptides.^49^

## Conclusions

In summary, we have demonstrated a generic strategy to prepare viral protein-based hybrid biomaterials mediated by a macromolecular glue that is inspired by mussel adhesives. AuNRs and SWCNTs were selected as typical nanomaterials to verify the concept of this macromolecular glue. Catechol modified negatively charged polymers show the capability to mediate the co-assembly of these nanometer-sized cargos and CCMV capsid proteins. This strategy provides a facile and general coating process compared with traditional methods.

In contrast to other commonly used ligands, such as, thiolate polymers and single-strand short DNA fragments, the feasibility of preparing PiBMAD with a diversity of other functional moieties makes this polymer a promising interfacial molecular glue for the construction of viral protein-based hybrid nanomaterials.

## Experimental

### 1. Material

All chemicals were purchased from Sigma-Aldrich and used as received unless stated. Deionized water used for buffers, reactions and dialysis media was of ultrapure quality (Milli-Q, 18.2 MΩ cm). Membrane filters (Spectra/Por® regenerated cellulose tubing) used for dialysis were purchased from Spectrum Laboratories, Inc., USA.

### 2. Methods

#### 2.1 UV-vis spectroscopy

UV-vis absorption spectra were acquired using a PerkinElmer Lambda 850 UV-vis spectrometer. Samples are prepared in a 1 cm quartz glass cuvette. Each recording cycle was performed within 44 s.

#### 2.2 Transmission electron microscopy (TEM)

TEM analysis was performed using a Philips CM300 microscope operating at 300 kV. A droplet of the samples was cast on a 200-mesh copper grid for 2 min before the excess solvent was blotted away using sterile paper. Samples were negatively stained by applying 5 μL 1% (w/v) uranyl acetate in MilliQ water onto the grid for 30 s and removed afterwards.

#### 2.3 Fast protein liquid chromatography (FPLC)

FPLC analysis was performed on a GE Healthcare ÄKTApurifier TM system equipped with a Superose 610/300 GL column from GE Healthcare and a fractionating device. Injection of 500 μL pre-filtered samples which are injected on a 24 mL superpose-6 column. Compound elution is monitored using a UV-vis spectrometer at 260 nm, 280 nm and 520 nm. Fractionation is collected separately.

#### 2.4 Dynamic light scattering (DLS)

DLS analysis was performed using a Nanotrac (Anaspec) instrument and Microtrac FLEX Operating software with a laser wavelength of 780 nm and a scattering angle of 90° at 25 °C. The observed size and standard deviation of nanoparticles were calculated by taking an average of 5 measurements.

#### 2.5 Nuclear magnetic resonance (NMR)

Proton NMR spectra of polymers were recorded using a Bruker 400 MHz NMR. Polymers were dissolved in dimethyl sulfoxide-d6 in concentrations of 0.5–1.0 mg mL^−1^ for ^1^H-NMR.

#### 2.6 Gel permeation chromatography (GPC)

The polydispersity of PiBMA was analyzed using a Waters e2695 Separations Module equipped with Agilent PLgel 5 μm MIXED-D 300 × 7.5 mmol L^−1^ column. DMF was used as an eluent, with molecular weights calibrated against linear standards of polyethylene glycol (PEG).

#### 2.7 Raman spectroscopy

Raman spectra of CNT samples were acquired using a custom-built Raman setup containing a 647 nm laser (30 mW). Calibrations were performed against an argon mercury light source as well as a white light source for further corrections. 50 μL of the sample was deposited on a glass slide and measured. From each measurement, an average of 100 spectra was used.

#### 2.8 Zeta potential

The zeta potential of gold nanorods with different ligands was measured at neutral pH, 25 °C with a Malvern Zetasizer Nano ZS ZEN3600, using a 633 nm laser.

#### 2.9 Cytotoxicity

HeLa cells were seeded in DMEM high glucose medium at 7.5 × 103 cells per well on 96-well plates and incubated at 37 °C in a humidified 5% CO_2_ containing atmosphere. Sample solutions were diluted in DMEM medium to the final concentration (5, 2.5, 1.25 nmol L^−1^ for AuNRs samples; 5, 10, 50 μg mL^−1^ for SWCNTs samples). After 24 h incubation, 100 μL of the sample solution or medium for the reference was added per well. The medium of the positive control was aspirated 30 min prior to the assay and replaced by 70% ethanol. After 24 h incubation, the medium was aspirated, and the cells were washed with HEPES buffer twice. Subsequently, 100 μL of resazurin sodium salt (440 mmol Lol^−1^ L^−1^) in HEPES was added and the cells were incubated for 24 h at 37 °C in a humidified 5% CO_2_ containing atmosphere. The fluorescence intensity was measured on a Tecan infinite m200 plate reader at an excitation and emission of 560/590 nm.

### 3. Sample preparation

#### 3.1. Synthesis of PiBMAD

1 g of PiBMA was dissolved in 50 mL aqueous solution of 2 mol L^−1^ NaOH. The solution was then hydrolyzed (Fig. S1a, ESI†) at 80 °C for 3 h, afterwards the solution turned clear. After cooling to room temperature, 15 mL of HCl (10 mol L^−1^) was added to precipitate PiBSA. The white, sticky polymer was then collected by short centrifugation and dissolved in 15 mL deionized water. In order to remove residual reagents, the solution was dialyzed against deionized water overnight using a 1 kD MWCO dialysis tube. The solution was then freeze dried (Labconco FreeZone 4.5) for 48 h to obtain 250–500 mg poly(isobutylene-*alt*-maleic acid) (PiBSA).

100 mg PiBSA was dissolved in 5 mL dimethylformamide (DMF) containing 1-ethyl-3-(3-dimethylaminopropyl)carbodiimide hydrochloride (EDC·HCl, 0.1 M) and *N* hydroxysuccinimide (NHS, 0.1 mol L^−1^). This mixture was stirred for 20 min. Meanwhile, a second solution was prepared of 47.4 mg dopamine·HCl (0.5 mol L^−1^) in 0.5 mL aqueous NaHCO3 (0.5 mol L^−1^) and Na_2_S_2_O_3_ (reducing agent, 0.1 mol L^−1^). The mixture was then added to the organic PiBSA solution and left to react overnight under mild magnetic stirring. The mixture was diluted 10× using deionized water and purified by dialysis against deionized water (1 kD MWCO dialysis tubing), refreshing the medium every 3 h for at least five times. The solution was then freeze-dried (Labconco FreeZone 4.5) for 48 h, after which the PiBMAD powder is collected.

#### 3.2. Encapsulation of PiBMAD by CCMV VLPs

PiBMAD was dissolved (6.0 mg mL^−1^) in pH 7.2 encapsulation buffer (50 mmol L^−1^ Tris, 200 mmol L^−1^ NaCl, 5 mmol L^−1^ MgCl_2_). 50 μL of this PiBMAD solution was added to 200 μL CP solution (6.0 mg mL^−1^, also in pH 7.2 encapsulation buffer). The mixture was gently shaken overnight at 4 °C.

#### 3.3. Preparation of CCMV-AuNRs

When extracting AuNRs from solution by means of centrifugation, to maximize the yield of AuNRs, samples were centrifuged as follows: A gold nanorod solution is centrifuged at 6000 g for 10 min. Then the pellet is collected, and the supernatant is centrifuged again at 8000 g for 10 min. The resulting pellet is collected, and the supernatant is again centrifuged at 10 000 g for 10 min. This is repeated once more at 14 000 g for 10 min, resulting in a total of four pellets after one round of centrifugation. All the centrifugation of AuNRs was performed in this manner, unless stated differently.

The ligand exchange of CTAB-coated gold nanorods with PiBMAD was based on general methods found in literature,^50^ where (poly)dopamine coatings are applied to gold nanorods. However, since oxidation of dopamine is undesired in our case, pH was lowered to 7.5 and a small amount of reducing agent (Na_2_S_2_O_3_) was included. Briefly, 1 mL CTAB-AuNRs (optical density = 0.95) from stock solution was centrifuged at 20 °C. The pellet was suspended in 2 mL Tris buffer (10 mmol L^−1^, pH 7.5) containing PiBMAD (0.5–4.0 mg mL^−1^ or 50–400 equiv. by weight conc.) and 5 mmol L^−1^ Na_2_S_2_O_3_. The nanorod suspension was then ultrasonicated for 30 min to accelerate displacement of the CTAB bilayer from the surface and subsequently incubated overnight at 4 °C. PiBMAD coated AuNRs were pelleted by centrifugation at 20 °C and resuspended in 1 mL deionized water. UV-vis-NIR absorption spectra and zeta potentials were recorded to confirm the presence of gold (SPR peaks) and polymer respectively.

Subsequently 1 mL PiBMAD-AuNRs were centrifuged once at 20 °C. The pellets were suspended and concentrated to OD ≈ 2–2.5 in 200 μL pH 6.8 cold encapsulation buffer (50 mmol L^−1^ Tris, 200 mmol L^−1^ NaCl and 5 mmol L^−1^ MgCl_2_). CP solution from storage, was freshly dialyzed to cold pH 6.8 encapsulation buffer, and diluted to a concentration of 6.0 mg mL^−1^. The PiBMAD-AuNRs and CP solution were then mixed in 1 : 1 volume ratio and gently stirred overnight at 4 °C. Note that due to the difficult purification of the nanorods after ligand exchange, there is still free PiBMAD in the nanorod solution, which is able to induce the formation of spherical VLPs. Hence an excess amount of CP is applied. After encapsulation CCMV-AuNRs were purified by FPLC. The collected fractions could be further purified by centrifugation at 6000 g for 10 min and were analyzed using UV-vis-NIR and TEM.

#### 3.4. Preparation of CCMV-SWCNTs

The dispersion of carbon nanotubes in aqueous media is a challenging process. Commonly used (ionic) surfactants, stated in decreasing order of dispersion quality,^51^ are sodium dodecyl sulfate (SDS), lithium dodecyl sulfate (LDS), sodium dodecylbenzene sulfonate (SBDS), tetradecyl trimethyl ammounium bromide (TTAB) and sodium cholate (SC). Dispersion generally occurs during long periods of ultrasonication, when more and more surfactant molecules are between bundled CNTs, ultimately breaking them free.^52^ Dispersion results seemed very inconsistent and the concentration ratio between SDS and dispersed CNT seem vary between 1.25 and 2000.^53^ In this research, amide functionalized SWCNT were used, with bundle dimensions of 4–6 × 1000 nm (commercial specifications). 0.5 mg of black SWCNT powder was added to 1 mL aqueous SDS (10 mg mL^−1^). The solution was then ultrasonicated in an ice bath for 3 h. After sonication, the SWCNT solution was centrifuged at 16 000 g for 1 h, precipitating all undispersed and amorphous carbon. The supernatant, containing SDS-SWCNTs was collected, and the presence of carbon nanotubes was investigated using Raman spectroscopy and TEM. Then 2 mg mL^−1^ PiBMAD solution in pH 7.5 Tris (20 mmol L^−1^, 10 mmol L^−1^ Na_2_S_2_O_3_) was prepared and added to aqueous SDS-SWCNT in a 1 : 1 vol. ratio. The mixture was then ultrasonicated for 2–3 h and centrifuged at 16 000 g for 30 min to remove aggregated SWCNT. The supernatant was collected and dialyzed (12–14 kD MWCO) against two to five volumes of 300 mL pH 6.8 encapsulation buffer (50 mmol L^−1^ Tris, 200 mmol L^−1^ NaCl, and 5 mmol L^−1^ MgCl_2_) to remove free polymer. After dialysis, aggregated carbon nanotubes were sonicated for 30 min for potential redispersion and then centrifuged for 30 min at 16 000 g. Due to insufficient removal of the polymer during dialysis, the solution was washed at least six times using a 100 kD spin filter with pH 6.8 encapsulation buffer. The carbon nanotubes, mostly sticking to the filter (around 90%), are entered back in the solution in aggregated form by thoroughly flushing the filter with 100 μL encapsulation buffer using a pipette and could be redispersed by 5–20 min ultrasonication. Then PiBMAD-SWCNTs (100 μL pH 6.8 encapsulation buffer) were added to 10 μL CP (6 mg mL^−1^, pH 6.8 encapsulation buffer) and gently stirred overnight at 4 °C. Excess CP was removed by washing at least three times with deionized water using a 100 kD spin filter.

#### 3.5. Alternative ligands

Besides our designed polymer glue, two alternatives are proposed as templates for the encapsulation of gold nanorods: polyDOPA (PDA)^54,55^ and bis(*p*-sulfonatophenyl)phenylphosphine (BSPP).^23^

##### 3.5.1. PolyDOPA

For the growth of PDA films on gold nanorods, 1 mL CTAB-AuNRs from stock solution was centrifuged and suspended in 2 mL Tris pH 8.5 buffer containing 0.5 mg mL^−1^l-DOPA. The solution was ultrasonicated for 30 min to allow for film growth, where the thickness depends on the sonication time and concentration of l-DOPA. After sonication, the PDA-AuNRs could not be precipitated into pellet anymore, unless the pH of the solution was significantly lower than the pKa of the carboxylic group (≈ 2.3) of l-DOPA. Centrifugation at pH = 1.8 resulted in a pellet which was suspended into 300 μL pH 6.8 encapsulation buffer (50 mmol L^−1^ Tris, 200 mmol L^−1^ NaCl, 5 mmol L^−1^ MgCl_2_). The PDA-AuNRs were then added (1 : 1 vol.) to 6.0 mg mL^−1^ CP (pH 6.8 enc. buffer) and mildly stirred overnight at 4 °C. Samples were inspected using TEM.

##### 3.5.2. Bis(*p*-sulfonatophenyl)phenylphosphine (BSPP)

The capping of AuNRs by BSPP was performed according to the method of capping spherical gold nanoparticles (AuNPs) described by A. Liu *et al.* Briefly,^23^ 1 mL AuNRs from stock solution was centrifuged and the pellet was suspended into 2 mL aqueous BSPP (0.5–3.0 mg mL^−1^). The solution was mildly stirred overnight at room temperature. BSPP-AuNRs were then centrifuged and concentrated in 200 μL pH 6.8 encapsulation buffer (50 mmol L^−1^ Tris, 200 mmol L^−1^ NaCl, 5 mmol L^−1^ MgCl_2_). A solution of freshly dialyzed CCMV CP into pH 6.8 encapsulation buffer was then diluted to 6 mg mL^−1^ and added to the BSPP-AuNR solution in a 1 : 1 volume ratio. The solution was then mildly stirred overnight at 4 °C. CCMV-BSPP-AuNRs were then purified by size exclusion chromatography (FPLC), the collected gold fractions were analyzed with TEM and UV-vis-NIR. The CCMV coated nanorods could be concentrated by centrifugation at 6000 g for 10 min at 4 °C and resuspension of the pellet.

Attempts to coat SWCNTs with BSPP and PDA were made. The coating of SWCNTs with BSPP and PDA were performed nearly identical to the method described previously for AuNRs. But BSPP was added in deionized water instead of tris buffer. For l-DOPA the following modifications were applied: a slightly elevated pH of 8.5 and the exclusion of Na_2_S_2_O_3_ to facilitate oxidation and a shorter ultrasonic treatment (1 h instead of 3 h) to limit film growth.

## Author contributions

The manuscript was written through contributions of all authors. All authors have given approval to the final version of the manuscript.

## Conflicts of interest

There are no conflicts to declare.

## Supplementary Material

TB-011-D2TB02688K-s001

## References

[cit1] Minten I. J., Claessen V. I., Blank K., Rowan A. E., Nolte R. J. M., Cornelissen J. J. L. M. (2011). Catalytic capsids: the art of confinement. Chem. Sci..

[cit2] Liu A., de Ruiter M. V., Zhu W., Maassen S. J., Yang L., Cornelissen J. J. L. M. (2018). Compartmentalized Thin Films with Customized Functionality via Interfacial Cross-linking of Protein Cages. Adv. Funct. Mater..

[cit3] Qiu G., Gai Z., Tao Y., Schmitt J., Kullak-Ublick G. A., Wang J. (2020). Dual-Functional Plasmonic Photothermal Biosensors for Highly Accurate Severe Acute Respiratory Syndrome Coronavirus 2 Detection. ACS Nano.

[cit4] Cheng Q., Wei T., Farbiak L., Johnson L. T., Dilliard S. A., Siegwart D. J. (2020). Selective organ targeting (SORT) nanoparticles for tissue-specific mRNA delivery and CRISPR-Cas gene editing. Nat. Nanotechnol..

[cit5] Li H., Wang X., Ohulchanskyy T. Y., Chen G. (2021). Lanthanide-Doped Near-Infrared Nanoparticles for Biophotonics. Adv. Mater..

[cit6] Tajoli F., Dengo N., Mognato M., Dolcet P., Lucchini G., Faresin A., Grunwaldt J. D., Huang X., Badocco D., Maggini M., Kübel C., Speghini A., Carofiglio T., Gross S. (2020). Microfluidic Crystallization of Surfactant-Free Doped Zinc Sulfide Nanoparticles for Optical Bioimaging Applications. ACS Appl. Mater. Interfaces.

[cit7] Zinelli R., Soni S., Cornelissen J. J. L. M., Michel-Souzy S., Nijhuis C. A. (2023). Charge transport across proteins inside proteins: Tunneling across encapsulin protein cages and the effect of cargo proteins. Biomolecules.

[cit8] Liu A., Traulsen C. H. H., Cornelissen J. J. L. M. (2016). Nitroarene Reduction by a Virus Protein Cage Based Nanoreactor. ACS Catal..

[cit9] Tejeda-Rodriguez J. A., Nunez A., Soto F., Garcia-Gradilla V., Cadena-Nava R., Wang J., Vazquez-Duhalt R. (2019). Virus-Based Nanomotors for Cargo Delivery. ChemNanoMat.

[cit10] Xue F., Cornelissen J. J. L. M., Yuan Q., Cao S. (2023). Delivery of MicroRNAs by plant virus-based nanoparticles to functionally alter the osteogenic differentiation of human mesenchymal stem cells. Chin. Chem. Lett..

[cit11] Wu Y., Yang H., Jeon Y.-J., Lee M.-Y., Li J., Shin H.-J. (2014). Surface modification of cowpea chlorotic mottle virus capsids via a copper(I)-catalyzed azide-alkyne cycloaddition (CuAAC) reaction and their adhesion behavior with HeLa cells. Biotechnol. Bioprocess Eng..

[cit12] Malay A. D., Miyazaki N., Biela A., Chakraborti S., Majsterkiewicz K., Stupka I., Kaplan C. S., Kowalczyk A., Piette B. M. A. G., Hochberg G. K. A., Wu D., Wrobel T. P., Fineberg A., Kushwah M. S., Kelemen M., Vavpetič P., Pelicon P., Kukura P., Benesch J. L. P., Iwasaki K., Heddle J. G. (2019). An ultra-stable gold-coordinated protein cage displaying reversible assembly. Nature.

[cit13] Schoonen L., van Hest J. C. M. (2018). Modification of CCMV nanocages for enzyme encapsulation. Methods Mol. Biol..

[cit14] Sinn S., Yang L., Biedermann F., Wang D., Kübel C., Cornelissen J. J. L. M., De Cola L. (2018). Templated Formation of Luminescent Virus-like Particles by Tailor-Made Pt(II) Amphiphiles. J. Am. Chem. Soc..

[cit15] Sun H., Luo Q., Hou C., Liu J. (2017). Nanostructures based on protein self-assembly: From hierarchical construction to bioinspired materials. Nano Today.

[cit16] Liu Q., Shaukat A., Kyllönen D., Kostiainen M. A. (2021). Polyelectrolyte Encapsulation and Confinement within Protein Cage-Inspired Nanocompartments. Pharmaceutics.

[cit17] Timmermans S. B. P. E., Ramezani A., Montalvo T., Nguyen M., van der Schoot P., van Hest J. C. M., Zandi R. (2022). The Dynamics of Viruslike Capsid Assembly and Disassembly. J. Am. Chem. Soc..

[cit18] Oliveira S. F., Bisker G., Bakh N. A., Gibbs S. L., Landry M. P., Strano M. S. (2015). Protein functionalized carbon nanomaterials for biomedical applications. Carbon.

[cit19] Zhang W., Patel K., Schexnider A., Banu S., Radadia A. D. (2014). Nanostructuring of Biosensing Electrodes with Nanodiamonds for Antibody Immobilization. ACS Nano.

[cit20] Comellas-Aragonès M., de la Escosura A., Dirks A. J., van der Ham A., Fusté-Cuñé A., Cornelissen J. J. L. M., Nolte R. J. M. (2009). Controlled Integration of Polymers into Viral Capsids. Biomacromolecules.

[cit21] de Ruiter M. V., Overeem N. J., Singhai G., Cornelissen J. J. L. M. (2018). Induced Förster resonance energy transfer by encapsulation of DNA-scaffold based probes inside a plant virus based protein cage. J. Phys.: Condens. Matter.

[cit22] Schoonen L., Maassen S., Nolte R. J. M., van Hest J. C. M. (2017). Stabilization of a Virus-Like Particle and Its Application as a Nanoreactor at Physiological Conditions. Biomacromolecules.

[cit23] Liu A., Verwegen M., de Ruiter M. V., Maassen S. J., Traulsen C. H. H., Cornelissen J. J. L. M. (2016). Protein Cages as Containers for Gold Nanoparticles. The. J. Phys. Chem. B.

[cit24] Wu Y., Cao S., Alam M. N. A., Raabe M., Michel-Souzy S., Wang Z., Wagner M., Ermarkova A., Cornelissen J. J. L. M., Weil T. (2010). Fluorescent nanodiamonds encapsulated by Cowpea Chlorotic Mottle Virus (CCMV) proteins for intracellular 3D-trajectory analysis. J. Mater. Chem. B.

[cit25] Lee H., Dellatore S. M., Miller W. M., Messersmith P. B. (2007). Mussel-Inspired Surface Chemistry for Multifunctional Coatings. Science.

[cit26] Zhu W., Peck Y., Iqbal J., Wang D. A. (2017). A novel DOPA-albumin based tissue adhesive for internal medical applications. Biomaterials.

[cit27] Xu J., Soliman G. M., Barralet J., Cerruti M. (2012). Mollusk glue inspired mucoadhesives for biomedical applications. Langmuir.

[cit28] Fullenkamp D. E., He L., Barrett D. G., Burghardt W. R., Messersmith P. B. (2013). Mussel-inspired histidine-based transient network metal coordination hydrogels. Macromolecules.

[cit29] Yang L., Kong J., Zhou D., Ang J. M., Phua S. L., Yee W. A., Liu H., Huang Y., Lu X. (2014). Transition-metal-ion-mediated polymerization of dopamine: mussel-inspired
approach for the facile synthesis of robust transition-metal nanoparticle-graphene hybrids. Chemistry.

[cit30] Wang B., Jeon Y., Bhang S. H., Kim J. (2017). Bioinspired dopamine-conjugated polyaspartamide as a novel and versatile adhesive material. eXPRESS Polym. Lett..

[cit31] Graña Suárez L., Verboom W., Huskens J. (2014). Cyclodextrin-based supramolecular nanoparticles stabilized by balancing attractive host–guest and repulsive electrostatic interactions. Chem. Commun..

[cit32] Ye Q., Zhou F., Liu W. (2011). Bioinspired catecholic chemistry for surface modification. Chem. Soc. Rev..

[cit33] Lee H., Dellatore S. M., Miller W. M., Messersmith P. B. (2007). Mussel-inspired surface chemistry for multifunctional coatings. Science.

[cit34] Minten I. J., Ma Y., Hempenius M. A., Vancso G. J., Nolte R. J. M., Cornelissen J. J. L. M. (2009). CCMV capsid formation induced by a functional negatively charged polymer. Org. Biomol. Chem..

[cit35] Zhou T., Yu M., Zhang B., Wang L., Wu X., Zhou H., Du Y., Hao J., Tu Y., Chen C., Wei T. (2014). Inhibition of Cancer Cell Migration by Gold Nanorods: Molecular Mechanisms and Implications for Cancer Therapy. Adv. Funct. Mater..

[cit36] Zeng C., Rodriguez Lázaro G., Tsvetkova I. B., Hagan M. F., Dragnea B. (2018). Defects and Chirality in the Nanoparticle-Directed Assembly of Spherocylindrical Shells of Virus Coat Proteins. ACS Nano.

[cit37] Durán-Meza A. L., Escamilla-Ruiz M. I., Segovia-González X. F., Villagrana-Escareño M. V., Vega-Acosta J. R., Ruiz-Garcia J. (2020). Encapsidation of Different Plasmonic Gold Nanoparticles by the CCMV capsid proteins. Molecules.

[cit38] Jung H. S., Cho K. J., Seol Y., Takagi Y., Dittmore A., Roche P. A., Neuman K. C. (2018). Polydopamine encapsulation of fluorescent nanodiamonds for biomedical applications. Adv. Funct. Mater..

[cit39] Trapaidze A., D'Antuono M., Fratzl P., Harrington M. J. (2018). Exploring mussel byssus fabrication with peptide-polymer hybrids: Role of pH and metal coordination in self-assembly and mechanics of histidine-rich domains. Eur. Polym. J..

[cit40] Hu P., Zhang J., Wen Z., Zhang C. (2011). Network single-walled carbon nanotube biosensors for fast and highly sensitive detection of proteins. Nanotechnology.

[cit41] Barone P. W. ; Jeng E. S.; Heller D. A. and Strano M. S., Biosensors based on single-walled carbon nanotube near-infrared fluorescence, John Wiley & Sons Ltd., 2007, pp. 843–854

[cit42] Cid C. C., Riu J., Maroto A., Rius F. X. (2010). Biosensors based on carbon nanotube-network field-effect transistors. Methods Mol. Biol..

[cit43] Lay C. L., Liu J., Liu Y. (2011). Functionalized carbon nanotubes for anticancer drug delivery. Expert Rev. Med. Devices.

[cit44] Dieckmann G. R., Dalton A. B., Johnson P. A., Razal J., Chen J., Giordano G. M., Muñoz E., Musselman I. H., Baughman R. H., Draper R. K. (2003). Controlled Assembly of Carbon Nanotubes by Designed Amphiphilic Peptide Helices. J. Am. Chem. Soc..

[cit45] Ortiz-Acevedo A., Xie H., Zorbas V., Sampson W. M., Dalton A. B., Baughman R. H., Draper R. K., Musselman I. H., Dieckmann G. R. (2005). Diameter-Selective Solubilization of Single-Walled Carbon Nanotubes by Reversible Cyclic Peptides. J. Am. Chem. Soc..

[cit46] Zheng M., Jagota A., Semke E. D., Diner B. A., McLean R. S., Lustig S. R., Richardson R. E., Tassi N. G. (2003). DNA-assisted dispersion and separation of carbon nanotubes. Nat. Mater..

[cit47] Kus-Liśkiewicz M., Fickers P., Ben Tahar I. (2021). Int. J. Mol. Sci..

[cit48] de Ruiter M., van der Hee R. M., Driesen A. J. M., Keurhorst E. M., Hamid M., Cornelissen J. J. L. M. (2019). J. Controlled Rel..

[cit49] Hassani-Mehraban A., Creutzburg S., van Heereveld L., Kormelink R. (2015). BMC Biotechnol..

[cit50] Black K. C., Yi J., Rivera J. G., Zelasko-Leon D. C., Messersmith P. B. (2013). Polydopamine-enabled surface functionalization of gold nanorods for cancer cell-targeted imaging and photothermal therapy. Nanomedicine.

[cit51] Sun Z., Nicolosi V., Rickard D., Bergin S. D., Aherne D., Coleman J. N. (2008). Quantitative Evaluation of Surfactant-stabilized Single-walled Carbon Nanotubes: Dispersion Quality and Its Correlation with Zeta Potential. The. J. Phys. Chem. C.

[cit52] Ramos E., Pardo W. A., Mir M., Samitier J. (2017). Dependence of carbon nanotubes dispersion kinetics on surfactants. Nanotechnology.

[cit53] Duan W. H., Wang Q., Collins F. (2011). Dispersion of carbon nanotubes with SDS surfactants: a study from a binding energy perspective. Chem. Sci..

[cit54] Ding Y. H., Floren M., Tan W. (2016). Mussel-inspired polydopamine for bio-surface functionalization. Biosurf. Biotribology.

[cit55] Sajitha M., Vindhyasarumi A., Gopi A., Yoosaf K. (2015). Shape controlled synthesis of multi-branched gold nanocrystals through a facile one-pot bifunctional biomolecular approach. RSC Adv..

