# Supplementary material for: Construction of viral protein-based hybrid nanomaterials mediated by a macromolecular glue
Source: J Mater Chem B. 2023 Jun 6;11(33):7933–41. doi: 10.1039/d2tb02688k (PMC10448939; doi:10.1039/d2tb02688k)
Supplement: TB-011-D2TB02688K-s001 [file TB-011-D2TB02688K-s001.pdf]

Electronic Supplementary Material (ESI) for Journal of Materials Chemistry B.
This journal is © The Royal Society of Chemistry 2023

Supporting information for

Construction of Viral Protein-based Hybrid Nanomaterials Mediated by a Macromolecular Glue

Shuqin Cao,[†‡] Sandro Peeters,[†] Sandra Michel-Souzy, [†]Naomi Hamelmann,[†] Jos M. J. Paulusse,[†] Liulin Yang,*[,†§] Jeroen J. L. M. Cornelissen*[,†]

[†]Laboratory for Biomolecular Nanotechnology, MESA+ Institute for Nanotechnology, University of Twente, P.O. Box 217, 7500 AE, Enschede, The Netherlands.

[‡]State Key Laboratory of Oral Diseases, West China Hospital of Stomatology, Sichuan University, Chengdu 610041, China.

[§]College of Chemistry and Chemical Engineering, Xiamen University, 361005, Xiamen, China.

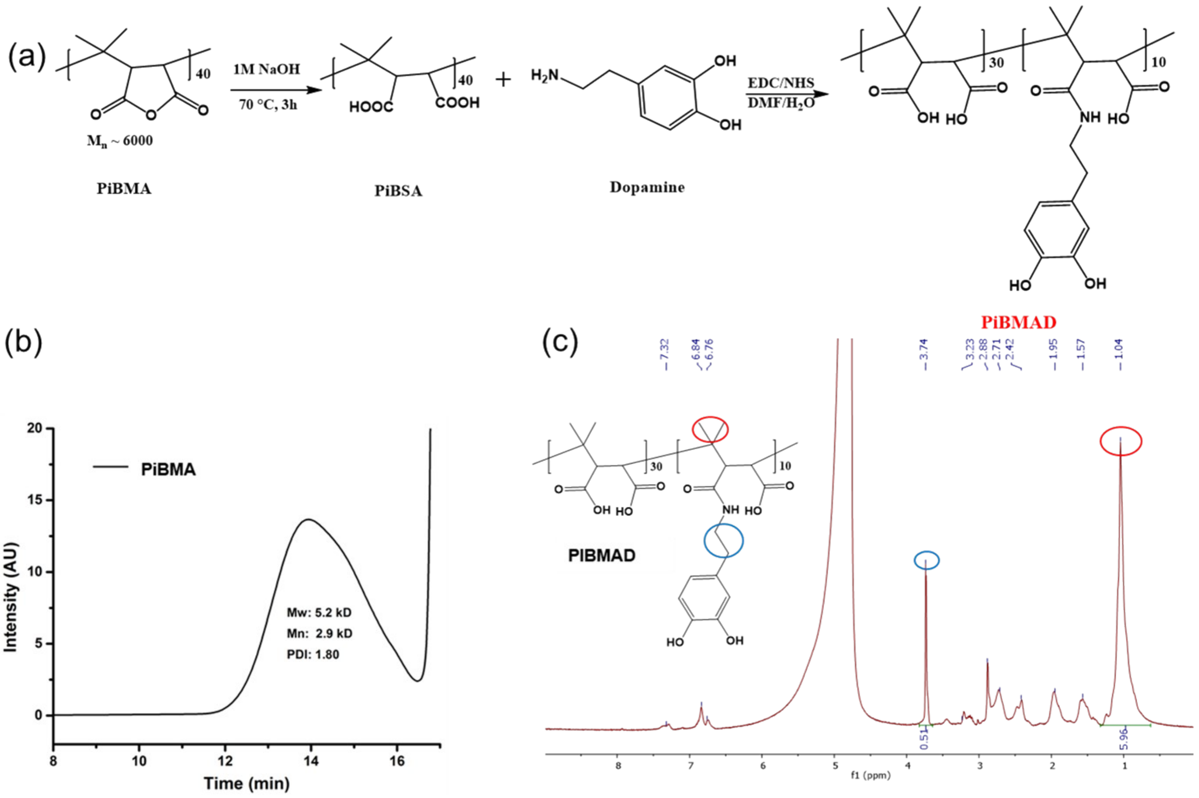

Figure S1. (a). Random functionalization to form PiBMAD; (b). GPC data of commercially available PiBMA; (c). ^{1}H-NMR characterization of PiBMAD.

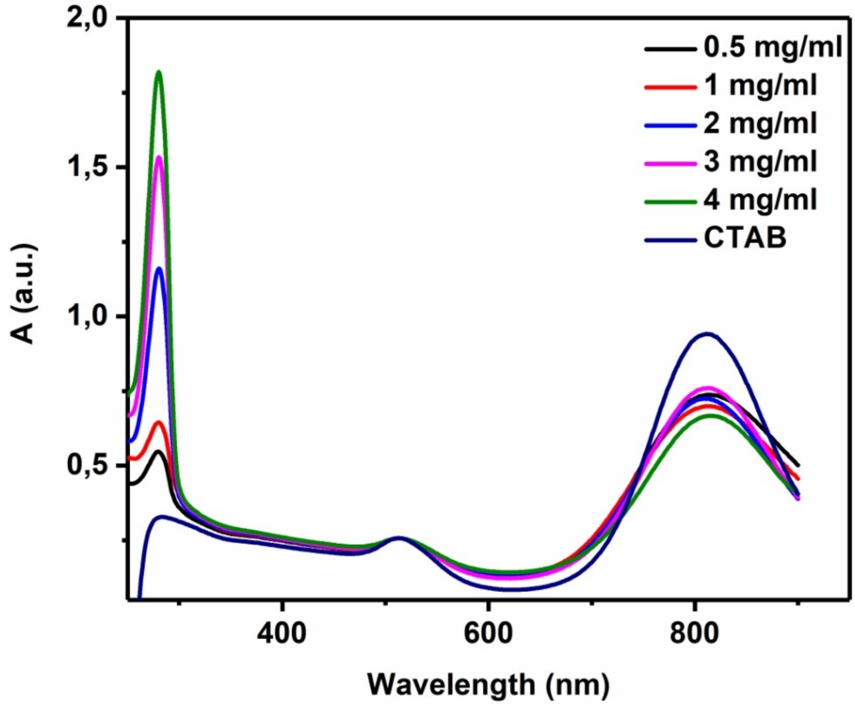

Figure S2. UV-vis data of PiBMAD-AuNRs formed at various polymer concentration, which indicated increased absorption of PiBMAD at λ = 280 nm at higher polymer concentration.

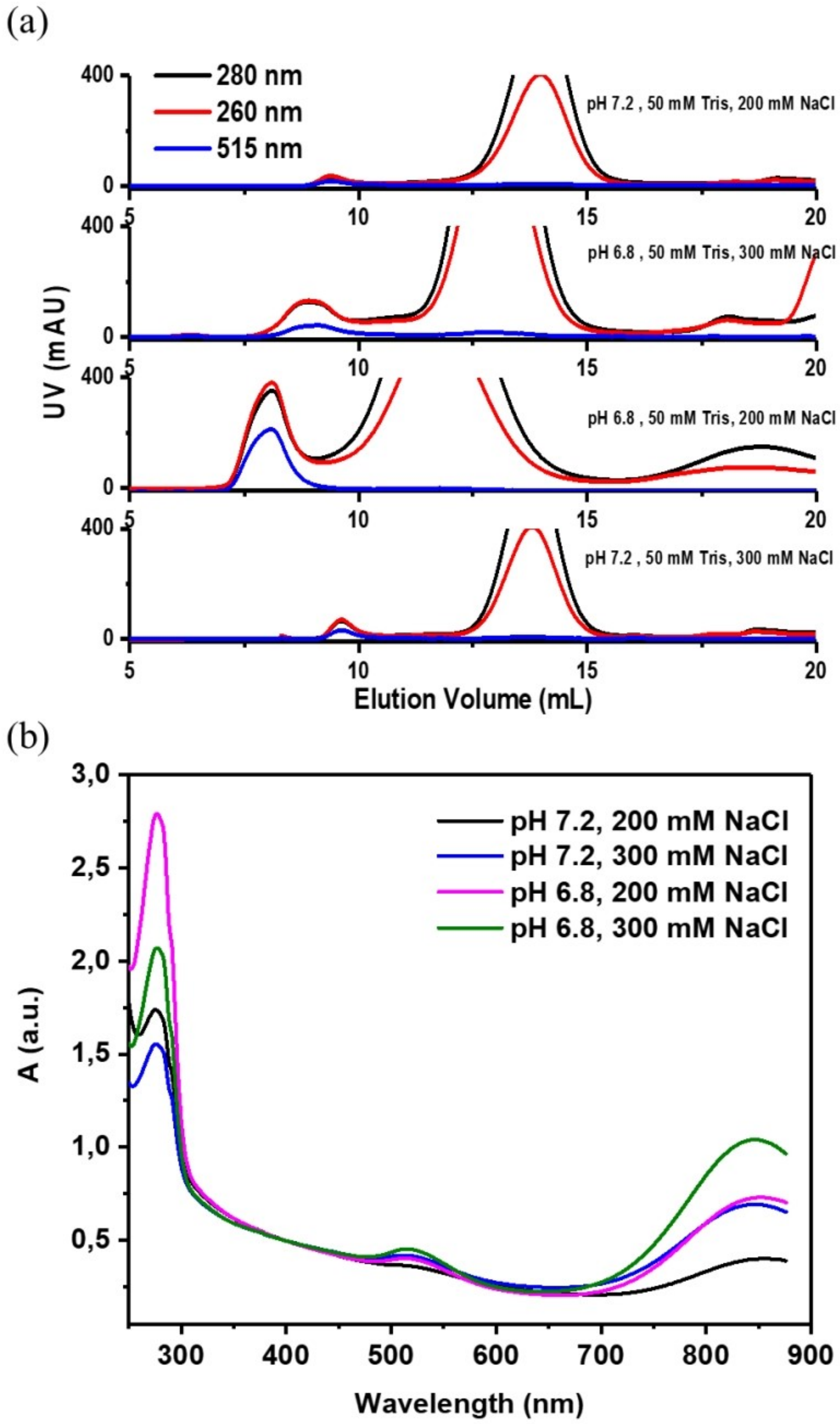

Figure S3. FPLC (a) and UV-vis (b) data of CCMV-AuNRs formed at various encapsulation buffers, which suggested thicker coating of CP on the surface of AuNRs at pH 6.8 encapsulation buffer (50 mmol/L tris, 200 mmol/L NaCl).

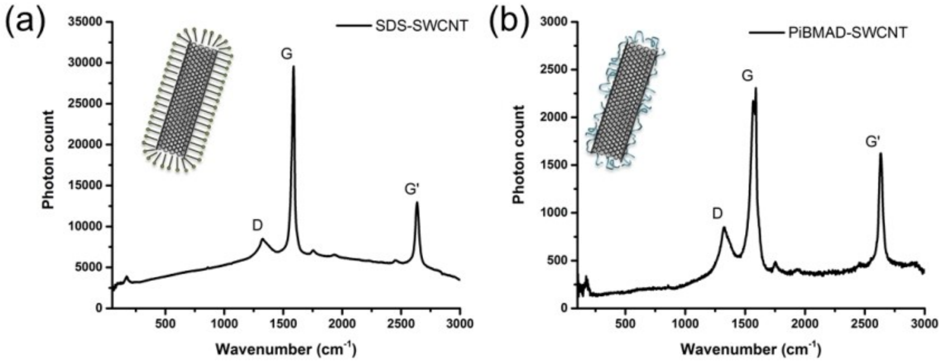

Figure S4. Raman shift of SWCNTS dispersed in water by (a) SDS and (b) PiBMAD

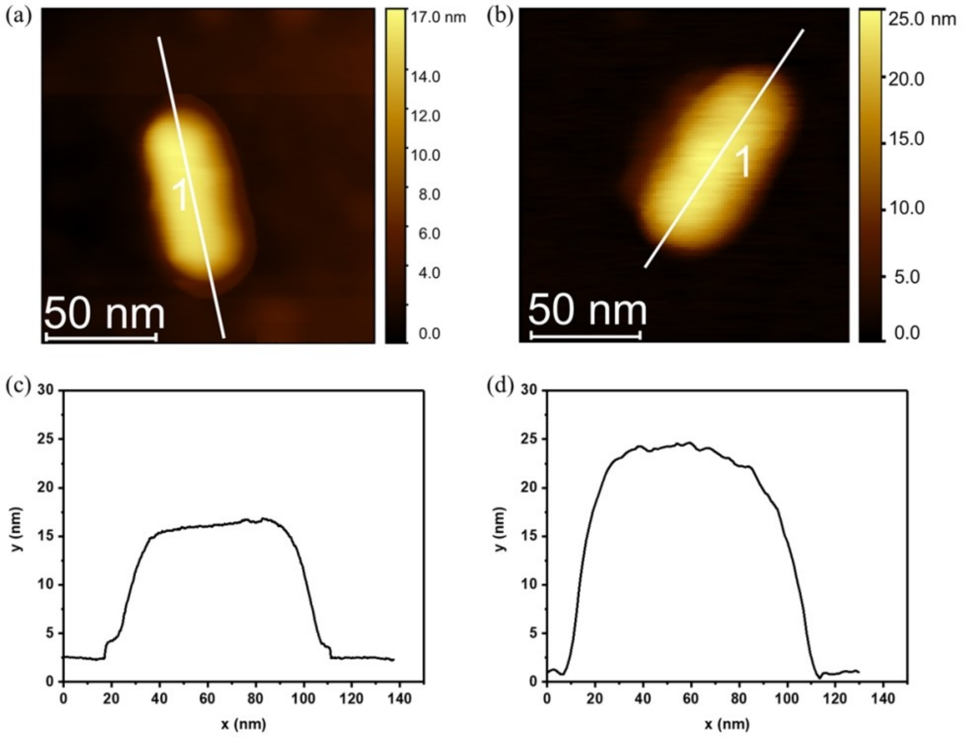

Figure S5. AFM morphology and height measurement of PiBMAD-AuNRs (a, c) and CCMV-AuNRs (b, d).

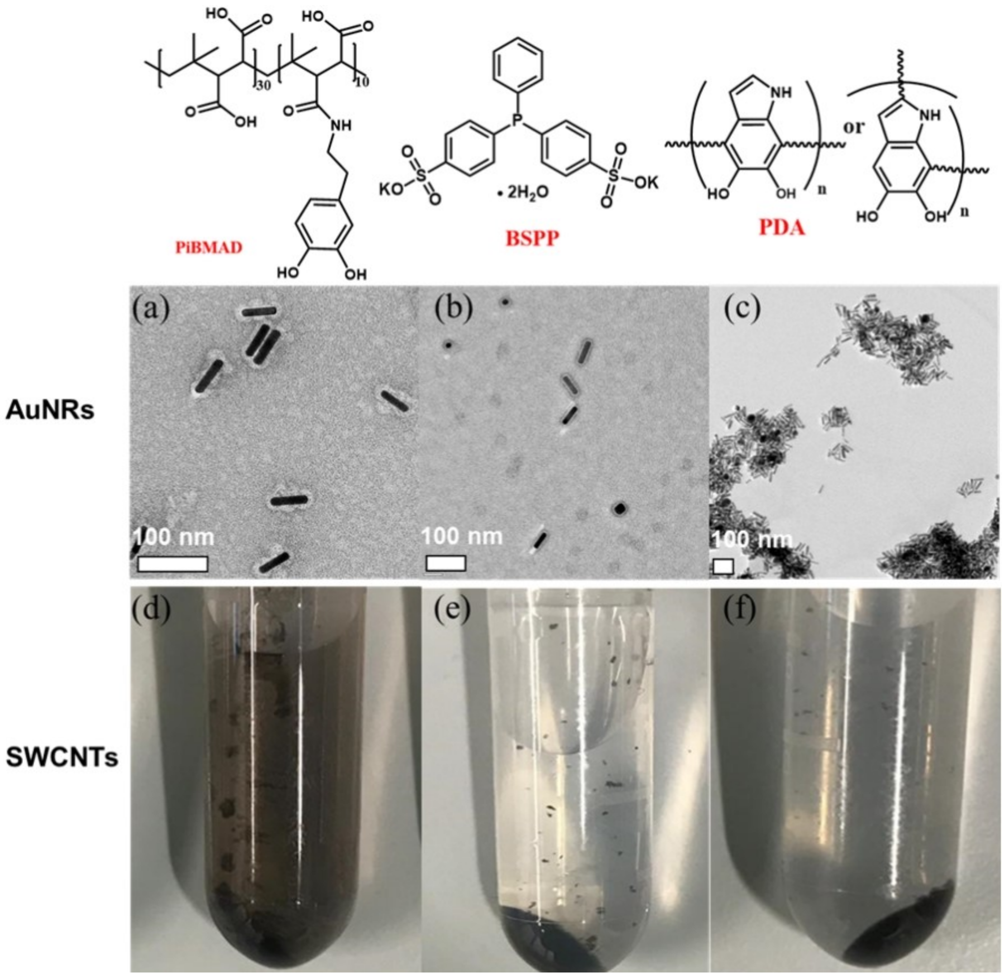

Figure S6. Alternative ligands were used as templates for coating of AuNRs (a-c) and SWCNTs (d-f).

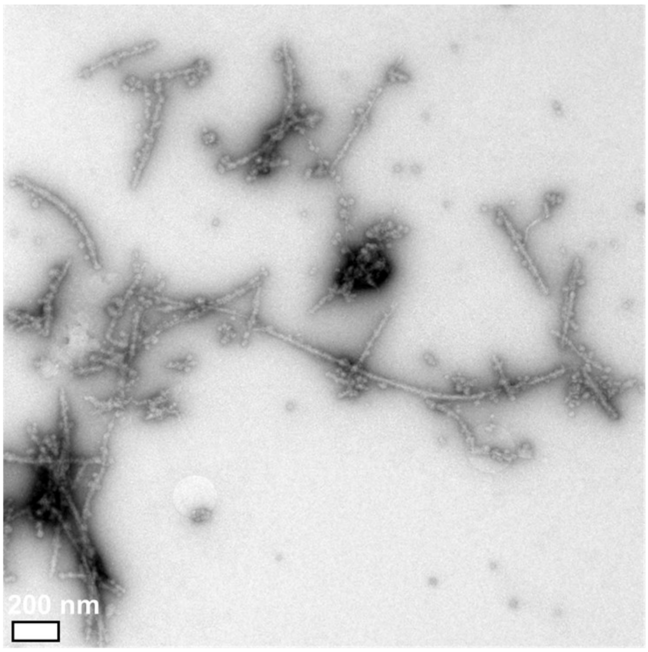

Figure S7. TEM image of CCMV-SWCNTs at lower magnification.

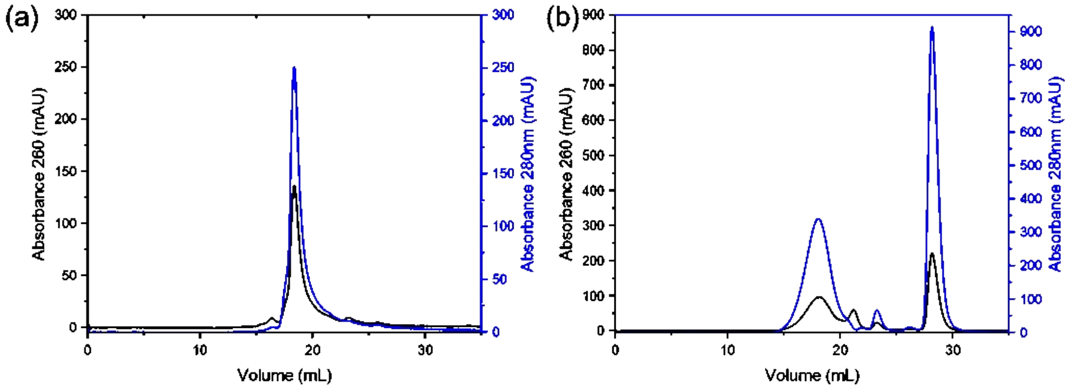

Figure S8. FPLC analysis of capsid protein (a) and PiBMAD (b). the elution volume of capsid protein is around 18.5 mL, while the PiBMAD has two main fractions which eluted out around 17.5 mL and 27.5 mL respectively which indicated aggregation of PiBMAD itself. But the A280 nm/A260 nm of these two analysis are different .
